# Influences of temperature and salinity on physicochemical properties and toxicity of zinc oxide nanoparticles to the marine diatom *Thalassiosira pseudonana*

**DOI:** 10.1038/s41598-017-03889-1

**Published:** 2017-06-16

**Authors:** Mana M. N. Yung, Kevin W. H. Kwok, Aleksandra B. Djurišić, John P. Giesy, Kenneth M. Y. Leung

**Affiliations:** 1 The Swire Institute of Marine Science and School of Biological Sciences, the University of Hong Kong, Pokfulam, Hong Kong China; 2 Department of Applied Biology and Chemical Technology, the Hong Kong Polytechnic University, Hung Hom, Hong Kong China; 3 Department of Physics, the University of Hong Kong, Pokfulam, Hong Kong China; 4 Department of Veterinary Biomedical Sciences and Toxicology Centre, University of Saskatchewan, Saskatoon, SK Canada; 5 Zoology Department, Center for Integrative Toxicology, Michigan State University, East Lansing, MI USA; 6 State Key Laboratory of Pollution Control and Resource Reuse, School of the Environment, Nanjing University, Nanjing, China; 7 Department of Biology, Hong Kong Baptist University, Kowloon Tong, Hong Kong China; 8 State Key Laboratory in Marine Pollution, City University of Hong Kong, Kowloon, Hong Kong China

## Abstract

Climate change is predicted to result in rising average temperature of seawater with more extreme thermal events, and frequent rainfalls in some coastal regions. It is imperative to understand how naturally mediated changes in temperature and salinity can modulate toxicity of chemical contaminants to marine life. Thus, this study investigated combined effects of temperature and salinity on toxicity of zinc oxide nanoparticles (ZnO-NPs) to the marine diatom *Thalassiosira pseudonana*. Because ZnO-NPs formed larger aggregations and released less zinc ions (Zn^2+^) at greater temperature and salinity, toxicity of ZnO-NPs to *T*. *pseudonana* was less at 25 °C than at 10 °C and less at 32 than 12 PSU. However, toxicity of ZnO-NPs was significantly greater at 30 °C, since *T*. *pseudonana* was near its upper thermal limit. Three test compounds, ZnO, ZnO-NPs and ZnSO_4_, displayed different toxic potencies and resulted in different profiles of expression of genes in *T*. *pseudonana*. This indicated that ZnO-NPs caused toxicity via different pathways compared to ZnSO_4_. Mechanisms of toxic action of the three compounds were also dependent on temperature and salinity. These results provide insights into molecular mechanisms underlying the responses of the diatom to ZnO-NPs and Zn^2+^ under various regimes of temperature and salinity.

## Introduction

Because zinc oxide nanoparticles (ZnO-NPs) can effectively absorb both ultraviolet radiation A and B^1^, they are extensively used in commercial sunscreen products. Sunscreen products can contain ZnO-NPs at concentrations as great as 25%^2^. Widespread use of ZnO-NPs in sunscreens will inevitably increase their releases into freshwater and coastal marine environments. In coastal waters, both temperature and salinity undergo natural, daily, seasonal and annual fluctuations, while they might also be affected by global climate change. The United Nations Intergovernmental Panel on Climate Change (IPCC) indicated in its Fifth Assessment Report that climate change would result in warming of oceans and changes in frequency and intensity of rainfall^3^. Under the scenario of greater greenhouse gas emissions, when compared to a baseline period of 1986–2005, mean ocean temperature in the top 100 m is predicted to increase by 2.0 °C by the end of the 21^st^ century^3^. Mean global precipitation is also predicted to increase with greater mean surface temperatures of oceans, while in some regions rainfall will be more frequent and more intense^3^. As a result, at the end of the 21^st^ century, coastal marine organisms, such as phytoplankton, will likely live in a warmer environment with more frequent periods of longer duration of lower salinity. Concurrently, such changes in temperature and salinity can potentially change the physicochemical behavior of ZnO-NPs in seawater, and hence alter their toxicity to marine organisms.

It has been reported that ZnO-NPs inhibited growth of the marine microalgae *Phaeodactylum tricornutum* and *Tetraselmis suecica* ^4, 5^ and reduced the chlorophyll *a* content in these species during 96-h exposure^4^. ZnO-NPs also induced reactive oxygen species (ROS) in the microalgae *T*. *suecica* and *Alexandrium minutum* ^4^. Higher temperature could increase toxic potency of ZnO-NPs to microalgae. The toxic potency of ZnO-NPs to the marine diatom *Skeletonema costatum* was greater at 28 °C than at 15 °C^6^. *S*. *costatum* was more tolerant to ZnO-NPs at 15 °C, possibly because it attained maximum nitrate assimilation at this temperature^7^. At a higher temperature (e.g., 28 °C), that is close to the upper thermal limit of *S*. *costatum*, the diatom would experience thermal stress and become less tolerant of chemical and/or physical stresses caused by ZnO-NPs^6^. A higher temperature might also enhance uptake of ZnO-NPs and zinc ions (Zn^2+^) in the diatom^6^. As a result, an increase in temperature can alter toxic potency of ZnO-NPs to the diatom.

In contrast, greater salinity resulted in lesser toxic potency of ZnO-NPs to the marine copepod *Tigriopus japonicus* ^8^, possibly attributable to lesser concentrations of dissolved Zn^2+^ being released from ZnO-NPs at greater salinities. Previously, Yung *et al*.^9^ reported that toxic potency of ZnO-NPs to the marine diatom *Thalassiosira pseudonana* was significantly less when salinity increased from 12 to 27 PSU. However, little is known about the interacting effects of temperature and salinity on the physicochemical properties and toxicity of nanoparticles such as ZnO-NPs to marine organisms. This study investigated combined effects of temperature and salinity on the physicochemical properties and toxicity of ZnO-NPs.

The marine diatom *T*. *pseudonana* was used as the test organism because it is distributed worldwide and can adapt to a wide range of temperatures and salinities^10^. Its genome sequence is available^11^, allowing study of molecular mechanisms of toxicity of ZnO-NPs to *T*. *pseudonana*. Three groups of genes in *T*. *pseudonana* relating to formation of frustules of silica, photosynthesis and oxidative stress were selected and analyzed in this study, which was designed to investigate accessory effects of temperature, salinity and concentration on physicochemical properties and toxicity of ZnO-NPs to *T*. *pseudonana*. The study also investigated molecular mechanisms of toxic action of ZnO-NPs to this species of diatom, under various combinations of temperature and salinity. The results of this study provide empirical information for predicting toxic potency of ZnO-NPs to diatoms at various combinations of salinity and temperature that might occur under various scenarios of global climate change.

## Results

### Influences of temperature and salinity on aggregation of ZnO and ZnO-NPs

**Figure 1:**
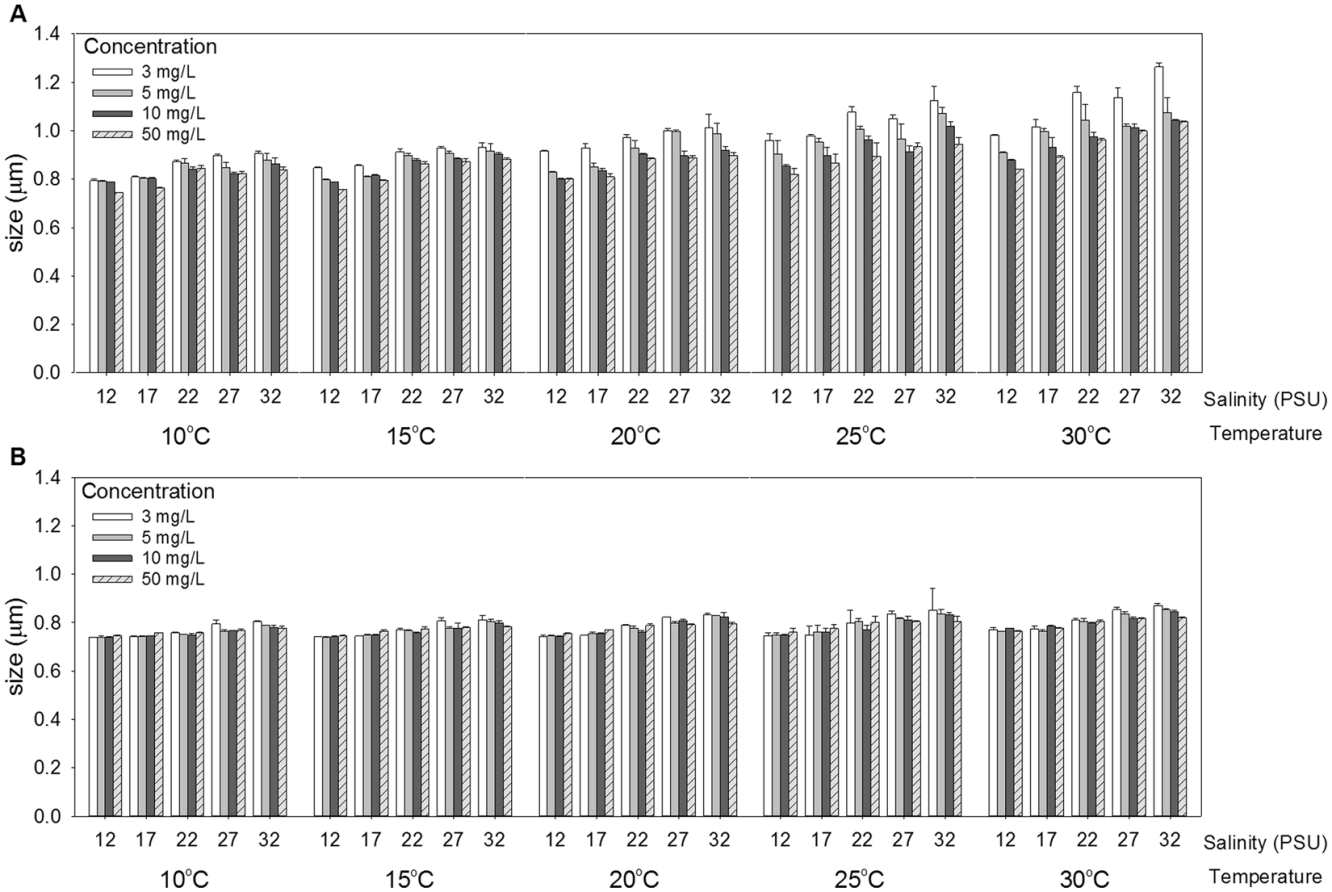
Mean size of aggregations after eight days of exposure to: (**A**) ZnO and (**B**) ZnO-NPs at several combinations of temperature, salinity and exposure concentration (mean and 95% confidence interval, *n* = 3). Sizes of aggregations of both particles at 0.5 and 1 mg/L were less than detection limit and were not shown.

Sizes of aggregations of ZnO and ZnO-NPs increased with increasing temperature and salinity (Fig. 1), but decreased with increasing exposure concentration of the chemical. Interactions among temperature, salinity, forms of Zn-containing chemicals and exposure concentration were statistically significant (Four-way ANOVA: *F*_48, 400_ = 2.50, *p* < 0.001). ZnO formed significantly larger aggregates than did ZnO-NPs (One-way ANOVA: *F*_1, 598_ = 492.41, *p* < 0.001).

### Influences of temperature and salinity on dissolution of ZnO and ZnO-NPs

**Figure 2:**
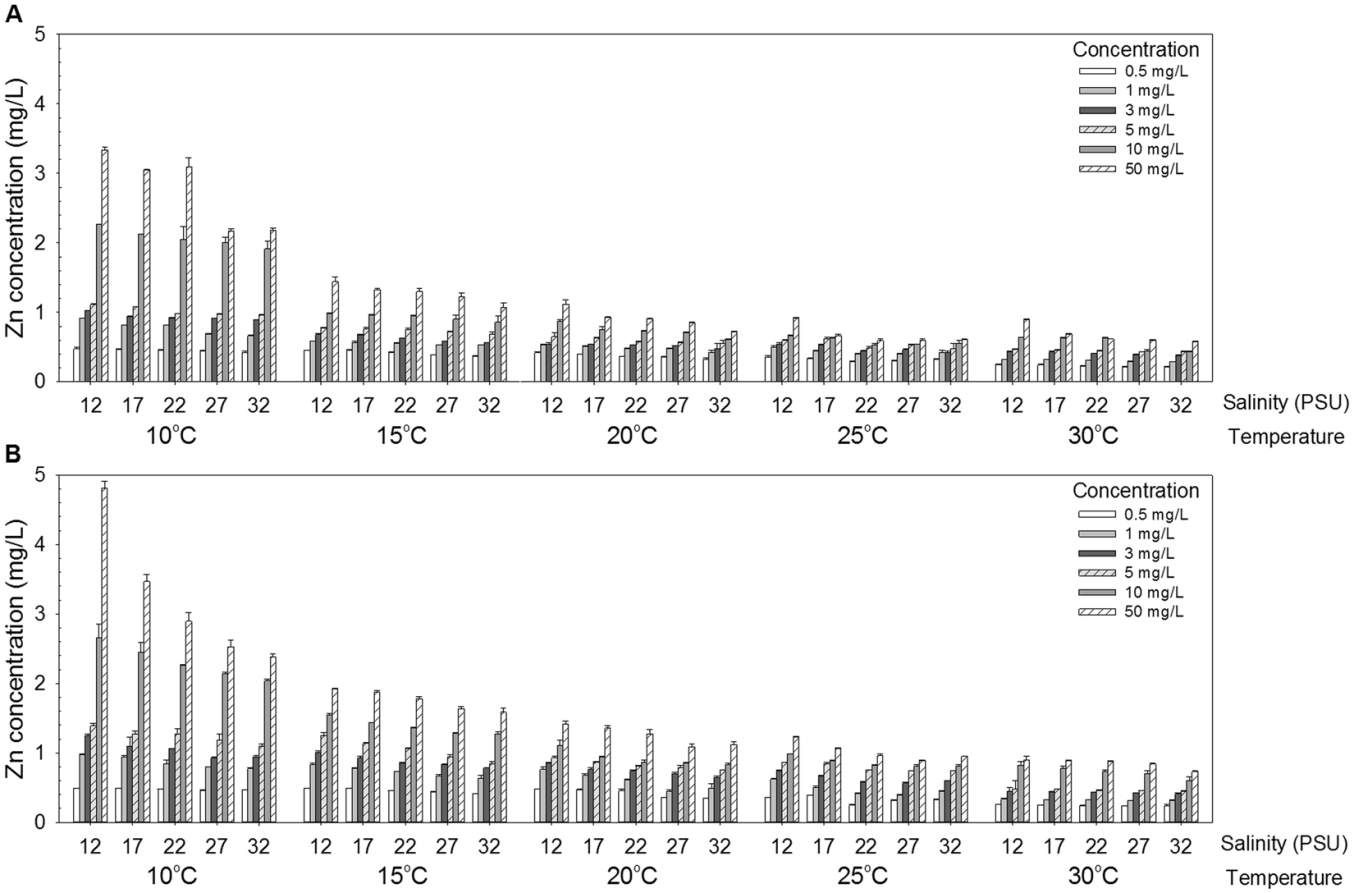
Mean dissolved concentration of Zn^2+^ after eight days of exposure in suspensions of: (**A**) ZnO and (**B**) ZnO-NPs at different combinations of temperature, salinity and exposure concentration (mean and 95% confidence interval, *n* = 3).

Concentrations of Zn^2+^ released by dissolution of ZnO and ZnO-NPs were inversely proportional to temperature and salinity (Fig. 2), but were directly proportional to the amounts of the compounds added. Drop in concentrations of Zn^2+^ across salinities was more obvious at lower than at higher temperatures. Interactions between temperature, salinity, form of Zn-containing compounds and concentration to which they were exposed were statistically significant (Four-way ANOVA: *F*_80, 600_ = 25.77, *p* < 0.001). More Zn^2+^ ions were dissolved from ZnO-NPs than from ZnO (One-way ANOVA: *F*_1, 898_ = 21.80, *p* < 0.001).

### Influences of temperature and salinity on the toxicity of the test chemicals

**Figure 3:**
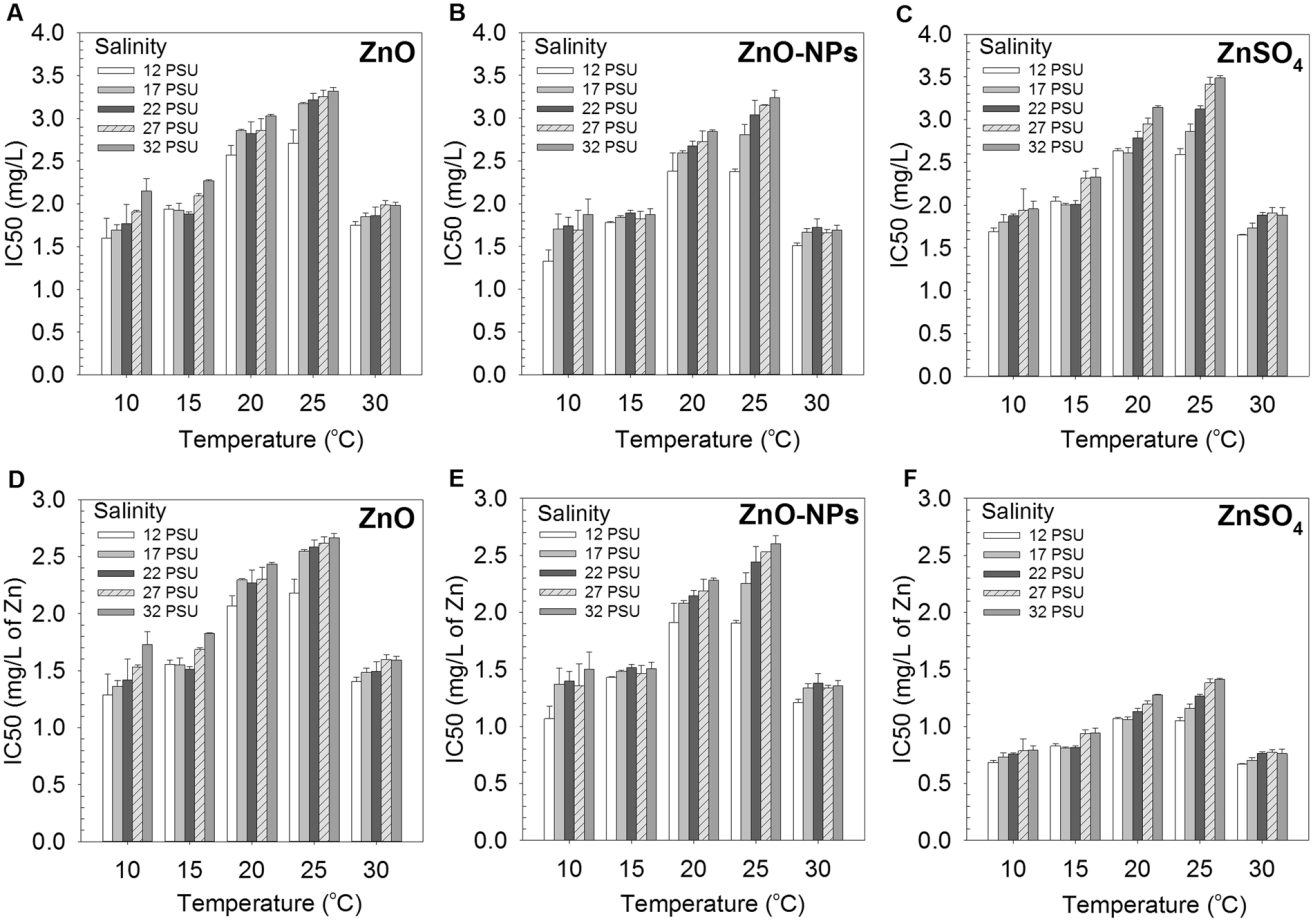
96-h median effect concentration (IC50) of three Zn-containing chemicals: ZnO, ZnO-NPs and ZnSO_4_ to the marine diatom *Thalassiosira pseudonana* at different combinations of temperature and salinity (mean and 95% confidence interval, *n* = 3). (**A**–**C**) IC50 values were calculated based on the particle concentrations; (**D–F**) IC50 values were calculated based on the total concentrations of Zinc (Zn). IC50 values of different treatment groups are given in Supplementary Information (SI: Table S1A–F).

Inhibition of growth of *T*. *pseudonana*, expressed as IC50 values calculated based on particle concentrations (Fig. 3A–C) and based on total Zn concentrations (Fig. 3D–F) is shown for ZnO, ZnO-NPs and ZnSO_4_. Inhibition of algal growth was lower at 25 °C, but it was greater at 10 °C ﻿a﻿nd 30 °C (Fig. 3). Toxic potencies of these three Zn-containing compounds were generally inversely proportional to salinity between 12 to 32 PSU, but such a trend was less obvious at 30 °C. Interactions between temperature and salinity were statistically significant on IC50 values of ZnO, ZnO-NPs and ZnSO_4_ calculated based on particle concentration (Two-way ANOVA: *F*_16, 200_ = 4.34, *p* < 0.001), with toxic potencies in decreasing order of: ZnO-NPs > ZnO and ZnSO_4_ (SNK post-hoc test, *p* < 0.05). However, there was no statistically significant interaction between temperature and salinity on IC50 values of ZnO, ZnO-NPs and ZnSO_4_ calculated based on total concentrations of Zn (Two-way ANOVA: *F*_16, 200_ = 0.19, *p* > 0.05). Temperature and salinity have individual effects on inhibition of algal growth based on total Zn concentration of the three chemicals (Temperature: *F*_4, 200_ = 35.2, *p* < 0.001; Salinity: *F*_4, 200_ = 2.65, *p* < 0.05), with toxic potencies in an order of: ZnSO_4_ > ZnO-NPs > ZnO.

### Influences of temperature and salinity on toxic mechanisms of the test chemicals

**Figure 4:**
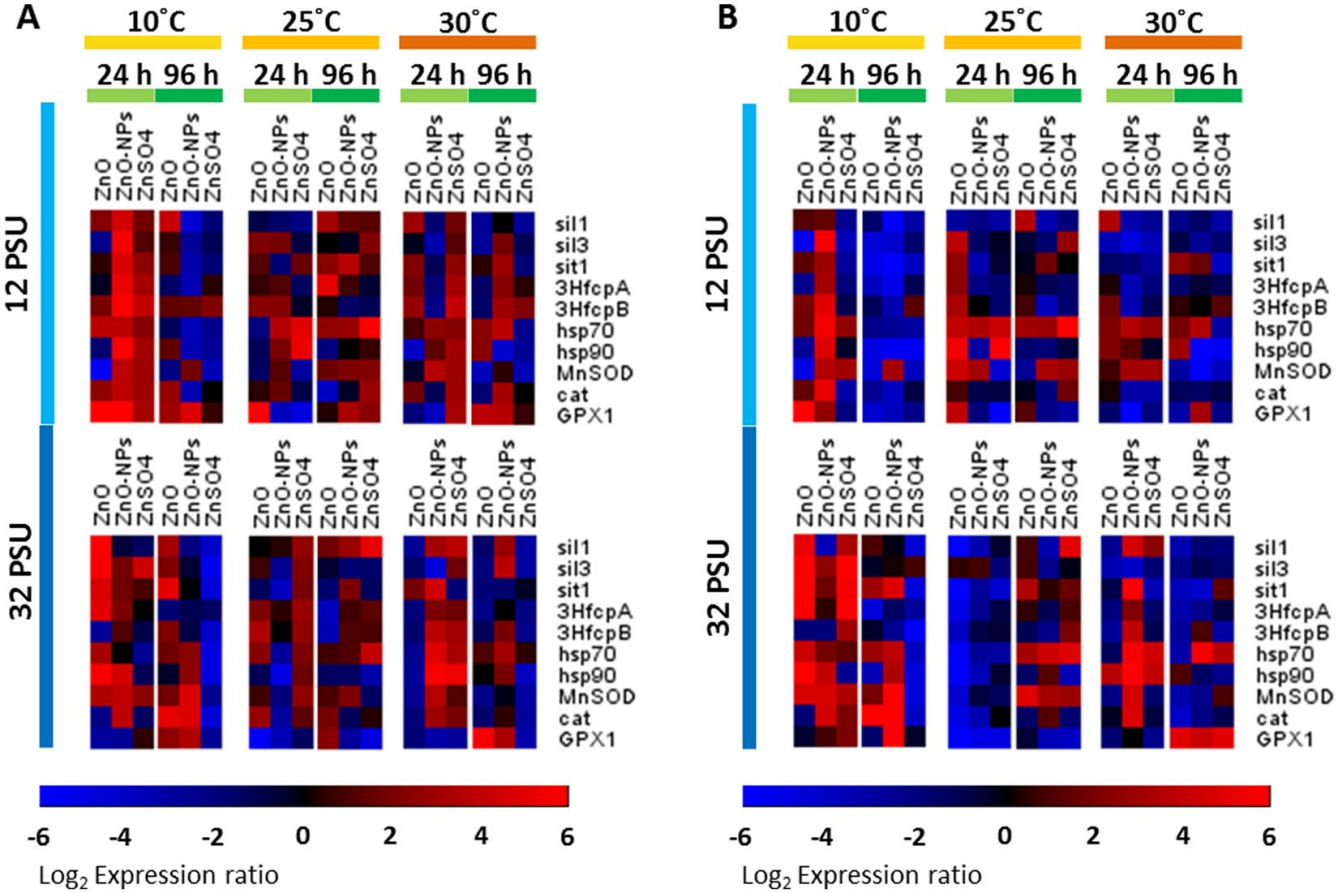
Heatmaps of differentially expressed genes of *T*. *pseudonana* exposed to ZnO, ZnO-NPs or ZnSO_4_ at (**A**) lesser (96-h IC20) and (**B**) greater (96-h IC50) concentrations under various combinations of temperature, salinity, and duration of exposure. *Gapdh* was used as a reference housekeeping gene to normalize the gene expression. Selected genes encoding for proteins are involved in formation of silica frustules of diatom (*sil1* and *sil3*), transportation of silica (*sit1*), photosynthesis (*3HfcpA* and *3HfcpB*), heat shock (*hsp70* and *hsp90*), and oxidative stress response (*MnSOD*, *cat* and *GPX1*).

The ten target genes were mostly up-regulated in *T*. *pseudonana* at 10 °C after 24 h of the exposure, whereas they were generally down-regulated at 30 °C after 96 h (Fig. 4). Patterns of differential expression were concentration-dependent as demonstrated by profiles of expression of genes between the lesser and greater concentration treatment. There were obvious differences in patterns of expression of genes among the three chemical treatments (ZnO, ZnO-NPs and ZnSO_4_), implying different modes of toxic action (Fig. 4). The ten target genes exhibited various responses to the three chemicals under different temperatures and salinities (SI, Figs S5 and S6). The genes encoding for proteins involved in formation of frustules of diatoms (*sil1* and *sil3*), transportation of silica (*sit1*) and oxidative stress (*MnSOD*, *cat* and *GPX1*) were the most responsive.

All five fixed factors, namely temperature, salinity, forms of Zn-containing chemicals, exposure concentration and time point exhibited significant statistical interactions on expression of genes of *T*. *pseudonana* (PERMANOVA: *pseudo* F = 47.64, *p* < 0.01). The three chemicals caused significantly different patterns of expression of genes in *T*. *pseudonana* (ANOSIM: *global* R = 0.023, *p* < 0.01). By comparing the *global* R values, temperature had a stronger effect on expressions of genes than did salinity (ANOSIM: temperature: *global* R = 0.059, *p* < 0.01; salinity: *global* R = 0.009, *p* < 0.01).

## Discussion

The results which are reported here, confirmed that physicochemical properties of ZnO and ZnO-NPs were dependent on both temperature and salinity. Higher temperature promoted aggregation of nanoparticles for two reasons^12^; first, the solution viscosity was less at higher temperature, so aggregation of particles was thus enhanced; second, interaction energies between nanoparticles was inversely proportional with temperature. Therefore, nanoparticles overcame the energy barrier more easily and aggregated more at higher temperatures. Zeta potentials of ZnO and ZnO-NPs were less at higher temperature such that the electrostatic repulsion force between particles became weaker, which would also promote aggregation (Fig. S2, SI). When salinity increases, the electric double layers of particles are compressed, the attractive van der Waals force dominates; and aggregation is enhanced^13^. Similar trends have been reported in other nanoparticles; aggregation was enhanced at greater temperature (CeO_2_; ZnO)^12, 14^ and ionic strength (CeO_2_; ZnO)^14, 15^.

ZnO-NPs undergoing aggregation will settle down by gravity^16^, and become less mobile in the water column^17, 18^. Sedimentation occurs readily when size of aggregation is greater than 1,000 nm^19^. Rate of sedimentation depends on the density of the particles, regardless of whether they are particles or aggregates, as well as the density of the fluid^20, 21^. ZnO-NPs take hours to aggregate and days to deposit in marine water^20^. Both temperature and salinity can also influence the rate of sedimentation. A rise in temperature increased the rate of sedimentation^22^. In seawater with high salinity, the rate of sedimentation was very high for TiO_2_, ZnO and CeO_2_ nanoparticles^23^. Aggregation and sedimentation of nanoparticles govern the fate and transportation of nanoparticles, and thus bioavailability and potential ecotoxicological impacts of these materials^20^.

Results of the study presented here demonstrated that dissolution of ZnO and ZnO-NPs decreased with increasing temperature and salinity. ZnO has a negative enthalpy of dissolution^24^, implied that dissolution of ZnO and ZnO-NPs releases heat. Higher temperature would, therefore, reduce dissolution of ZnO and ZnO-NPs^25^. Previous studies showed that solubility of ZnO was less than 1 mg/L at 35 °C while at 15 °C it was 3.5 mg/L^26^. At higher salinity, when released from ZnO or ZnO-NPs, Zn^2+^ can form complexes with the abundant anions (e.g., Cl^−^ and SO_4_^2−^) in seawater which would reduce the concentration of free Zn^2+^ ions^27^. Concentrations of dissolved zinc in the test suspension of ZnO were less than that in ZnO-NPs because ZnO has significantly larger sizes of particles (Fig. S1, SI), and thus a smaller surface-to-volume ratio than do ZnO-NPs.

The observed temperature-dependent relationship between potencies of chemicals is also commonly found in other marine organisms^28, 29^. In this study, *T*. *pseudonana* attained a maximum growth rate at 20–25 °C (Fig. S3, SI), which is consistent with results of studies with other organisms^10, 30^. Upper thermal limit for growth of *T*. *pseudonana* was close to 30 °C^31^. *T*. *pseudonana* was more susceptible to toxicity of ZnO-NPs at lower temperatures (e.g., 10 °C) possibly because more zinc ions were present at lower temperatures. It has been demonstrated that rate of protein synthesis per cell of *T*. *pseudonana* was less at lower temperature of 11 °C than at higher temperatures^32^. Cells of diatoms would have to increase cellular concentrations of ribosomal proteins to partially compensate for the reduced translation efficiency at lower temperatures^32^. Diatoms might allocate more energy to resist stress caused by colder temperatures and become less defensive to stress induced by ZnO-NPs and associated Zn^2+^ released to the medium.

At higher temperatures, concentrations of bioavailable Zn^2+^ ions were less, which resulted in lesser toxicity of ZnO-NPs at temperature in the range of 20 °C to 25 °C. Toxicity of ZnO-NPs was significantly greater at 30 °C, which is close to the upper thermal limit of *T*. *pseudonana* ^31^. First, based on the Q10 rule, higher temperature would enhance accumulation of metal ions by diatoms and lead to nutrient deficiency. When the marine diatom *Thalassiosira nordenskioeldii* was exposed to cadmium under thermal stress at 30.5 °C, enhanced accumulation of Cd was observed in diatom cells, which also exhibited weakened detoxification ability due to nitrogen deficiency and depletion of the antioxidant, glutathione^33^. Second, higher temperature can impair the photosynthetic performance of microalgae. Toxic effects of silver nanoparticles on two green algae *Chlorella vulgaris* and *Dunaliella tertiolecta* were enhanced due to deterioration of chlorophyll when temperature was raised from 25 °C to 31 °C^34^. With disruption of normal physiological function at higher temperatures, *T*. *pseudonana* would be more susceptible to toxic effects of ZnO-NPs and associated Zn^2+^ ions, despite the fact that lesser concentrations of Zn^2+^ ions were present. Third, there might be more ROS generated from ZnO-NPs at higher temperatures, which would result in greater toxicity. Toxicity of ZnO nano-fluid to two bacteria *Escherichia coli* and *Staphylococcus aureus* was greater at higher temperatures, with greater antibacterial activity at 42 °C compared to that at 25 °C and 37 °C for both bacteria^35^. It was suggested that at higher temperatures more ROS would be produced, which would result in greater toxicity to bacteria.

At greater salinities, due to complexation of zinc ions (Zn^2+^) with other anion present in seawater, amounts of bioavailable Zn^2+^ would be less^27^. The 96-h LC50s of ZnO-NPs for the marine copepod *Tigriopus japonicus* were 1.22 mg/L and 2.44 mg/L at salinities of 5 and 35 ppt, respectively^8^. After exposure to citrate-coated silver nanoparticles (5 mg/L) for 48 h, uptake of silver by embryos of the Atlantic killifish *Fundulus heteroclitus* was more than 2-fold greater in freshwater (0 PSU) than in brackish water (10 PSU)^36^. Consistent with the results presented here, results of another study showed that the 96-h IC50s of ZnO-NPs to *T*. *pseudonana* at 25 °C were 1.4 and 3.4 mg/L at 12 and 32 PSU, respectively^9^.

Previous studies have reported temperature and salinity have combined effects on growth of algae^37^, contents of photosynthetic pigments in algae^38^, survival time and growth of juvenile daphnia^39^, and survival and larval development of larval barnacles^40^. Temperature and salinity could also affect uptake of metals, such as copper, zinc and cadmium in submersed plants *Elodea Canadensis* and *Potamogeton natans*, with accumulation of metals directly proportional to temperature, but inversely proportional to salinity^41^. Comparatively, as shown in this study, growth of diatoms was more affected by temperature than by salinity. Temperature is a dominant accessory factor controlling the growth of aquatic organisms, such as microalgae, fish and cladocerans^10, 30, 42, 43^. In this study, 96-h IC50s of ZnO-NPs for *T*. *pseudonana* were 1.91 and 2.60 mg/L of Zn at 12 and 32 PSU at 25 °C, respectively. These values were to 1.07 and 1.50 mg/L of Zn at 12 and 32 PSU at a temperature of 10 °C, respectively. Toxic potencies were 1.21 and 1.36 mg/L of Zn at 12 and 32 PSU at 30 °C, respectively. The diatom *T*. *pseudonana* was more tolerant of ZnO-NPs at its optimum temperature (25 °C) and greater salinity (32 PSU), but it was more susceptible to chemical stress at extreme temperatures (10 °C and 30 °C) and lesser salinity (12 PSU).

Based on profiles of differentially expressed genes, when exposed to ZnO-NPs at greater salinity (i.e., 32 PSU) the diatom exhibited oxidative stress earlier at 10 °C than at 25 °C. This result was possibly due to more Zn^2+^ ions being available at lower temperature, while cold stress alone would lead to inhibition of growth and oxidative damage in algae^44^. When exposed to ZnSO_4_ at 10 °C, the diatom exhibited oxidative stress, impairing formation of frustules and decreased photosynthetic performance. Division of cells and rate of growth were also less. Exacerbated toxic effects of ZnO-NPs and ZnSO_4_ at lower temperature were related to effects of Zn^2+^ and lower temperature.

When *T*. *pseudonana* was exposed to ZnO-NPs at greater concentration (i.e., 96-h IC50) at the highest temperature (i.e., 30 °C) and at the greatest salinity (i.e., 32 PSU), expressions of genes indicated that oxidative stress was induced in the first 24 h as demonstrated by up-regulation of *hsp70*, *hsp90*, *MnSOD* and *cat* (Fig. 4B). Down-regulation of *sil1*, *sil3* and *sit1* after 96 h implied that formation of silica frustules of diatoms was affected by exposure to ZnO-NPs. Down-regulation of *3HfcpA* and *3HfcpB* was also observed after 96 h when the diatoms were exposed to ZnO-NPs at higher temperature and salinity, photosynthetic performance of diatoms was degraded. As suggested previously^35^, generation of ROS is expected to be greater at higher temperature. The observation of down-regulation of *3HfcpA* and *3HfcpB* during this study is consistent with the observation of lesser concentrations of photosynthetic pigments in algae at higher temperatures^34^. When *T*. *pseudonana* exposed to greater concentration of ZnSO_4_ at 30 °C and 32 PSU (Fig. 4B), up-regulation of *hsp70* and *hsp90* was observed at first 24 h, implying that the oxidative stress was induced in the diatoms. Down-regulation of *sil1*, *sil3*, *sit1*, *3HfcpA* and *3HfcpB* was also observed after 24 h, indicating that formation of silica frustule and photosynthetic performance of the diatoms were affected by exposure to ZnSO_4_. At higher temperatures, diatoms exposed to ZnSO_4_ were under combined effects of thermal and oxidative stress, resulting in inhibition of formation of cell walls and photosynthetic activity.

Based on expressions of genes, exposure to ZnO-NPs at higher temperatures and lesser salinity caused damage to frustules, inhibited photosynthesis and caused oxidative stress during earlier stage of exposure. Upon exposure to ZnSO_4_, diatoms first experienced oxidative stress and heat stress, then exhibited damage to frustules at lesser concentrations. When exposed to greater concentrations of ZnO-NPs and ZnSO_4_, *T*. *pseudonana* exhibited similar patterns of expression of genes. Severe damage was also observed at lower temperatures and lesser salinities, with similar patterns of expression of genes caused by exposure to ZnO-NPs or ZnSO_4_. Similar responses were likely due to more Zn^2+^ ions being available under those conditions. Overall, patterns of differential expression of genes at different combinations of temperature and salinity were related to availability of Zn^2+^ ions, interactions between surfaces of particles and surfaces of cells, and changes that they caused in the physiology of *T*. *pseudonana*.

At 30 °C and salinity of 12 PSU ZnO-NPs caused significant inhibition of growth of *T*. *pseudonana* and increased overall toxicity of ZnO-NPs. IPCC has predicted that there will be more extreme rainfall events and the average sea surface temperature will be likely increased by 2 °C by the end of this century under the projected greenhouse gas emission scenario. Because of the long-term heat transfer from sea surface to deeper ocean, the warming will continue even if emissions of greenhouse gases are reduced or kept constant^3^. Populations of marine diatoms, and hence the aquatic food chain, might be severely affected by extreme scenarios under the influence of climate change.

Given evidence of significant influences of temperature and salinity on the physicochemical properties and toxic potency of chemicals like ZnO-NPs, it is important to consider combined effects of multiple environmental stressors with a view to realizing the actual ecological impacts of chemical contaminants. However, this study did not include the carbon dioxide and pH into consideration; the ocean acidification will be an important aspect for inclusion in further studies for revealing the climate change impacts on chemical toxicity to primary producers.

## Methods

### Chemical preparation

ZnO-NPs as dry powders (20 nm; 99.5% purity; surface without modification), were purchased from Nanostructured & Amorphous Materials Inc. (New Mexico, USA) with specific surface area of 50 m^2^/g (manufacturer data). Zinc oxide (99.99% purity) and zinc sulphate (ZnSO_4_; >99.9% purity) were purchased from Sigma-Aldrich (St. Louis, MO, USA). TEM images of ZnO and ZnO-NPs are provided (Fig. S1). Briefly, particle size of ZnO (135 ± 9 nm; mean ± 95% confidence interval) was significantly larger than ZnO-NPs (27 ± 1 nm) (*t*_0.05 (2), 104_ = 23.28, *p* < 0.001) based on observations of 100 particles in TEM images. ZnO and ZnO-NPs suspensions at five test salinities (12, 17, 22, 27 and 32 PSU ± 0.5 PSU; pH 8.2 ± 0.1) and five temperatures (10, 15, 20, 25 and 30 ± 1 °C) were prepared in autoclaved, filtered artificial seawater (Supporting Information). Six test concentrations of 0.5, 1, 3, 5, 10, and 50 mg/L were used in physicochemical analyses and toxicity tests with diatoms.

### Physicochemical characterization of ZnO and ZnO-NPs

Bulk ZnO particles or ZnO-NPs in each of the 150 treatments (i.e., 5 temperatures × 5 salinities × 6 concentrations) were analysed for their size of aggregation, ion dissolution and zeta potential. All samplings and measurements were conducted in triplicate after eight days of stirring. A laser diffractometer (LS 13320 Series, Beckman Coulter Inc., Fullerton) was used to analyse the sizes of aggregations of ZnO and ZnO-NPs in each sample (50 mL each).

An inductively coupled plasma optical emission spectrometer (ICP-OES; ICP Optima 8300, Perkin Elmer, USA) was used to measure concentration of dissolved zinc in samples that was ablated from ZnO and ZnO-NPs. To remove nanoparticles, suspensions of ZnO and ZnO-NPs in each of the 150 treatments were filtered through 0.02-µm sterile syringe filters (Anotop 25, Whatman, England). Filtrates (8 mL each) were digested with 2% HNO_3_ and measured in triplicate. Zinc Pure AS calibration standard (1,000 mg/L dissolved in 2% HNO_3_), supplied from Perkin Elmer (Waltham, USA), was used for calibration and as reference. A blank treatment, without any addition of ZnO or ZnO-NPs, was used as the control to evaluate the background concentration of Zn. The limit of detection of ICP-OES for Zn is 1 µg/L.

A Delsa Nano C particle analyzer (Beckman Coulter Inc., Germany) was used to measure the zeta potential of ZnO and ZnO-NPs suspensions in the triplicate samples from each of the 150 treatments (5 mL each) (Fig. S2, SI).

### Diatom culture conditions and acclimation

The marine diatom *T*. *pseudonana* (CCMP 1335, Provasoli-Guillard National Centre for Marine Algae and Microbiota, USA) was originally cultured in autoclaved f/2 + Si medium^45^ at 25 ± 1 °C, 32 PSU (practical salinity unit), pH 8.2 ± 0.1 and a 14:10 h light: dark photoperiod inside an environmental chamber (Adaptis A350, Conviron, Canada) with a mean light intensity of 1160 lux (±80 lux; ±95% confidence interval; *n* = 20; LT Lutron LM-8000A, Taiwan). *T*. *pseudonana* was acclimated in autoclaved f/2 + Si medium from 32 PSU to each of the target salinities through a stepwise reduction of salinity by 2–3 PSU per day. After attaining the target salinity, diatoms were sub-cultured for three generations (i.e., 2 weeks) inside an environmental chamber. Diatoms were further acclimated to test temperatures by increasing or decreasing from 25 °C in a stepwise manner of 1 °C per day using a water bath system equipped with a chiller (Hailea, HC-300A, China) and a heater with a thermostat (Julabo GmbH, Germany). Diatoms were shaken regularly under a 14:10 h light: dark photoperiod with a mean light intensity of 1190 lux (±80 lux; ±95% confidence interval; *n* = 20; LT Lutron LM-8000A, Taiwan). Once the diatoms had attained the target temperature for at least 24 h, they were then cultured at each of the designated temperature and salinity regimes for three sub-cultured batches before the measurement of initial growth rate (Fig. S3, SI) and inhibition of algal growth.

### 96-h algal growth inhibition test

The 96-h algal growth inhibition test was conducted following OECD guidelines^46^. The experiment was conducted at five temperatures: 10, 15, 20, 25 or 30 °C with five salinities: 12, 17, 22, 27 or 32 PSU. At each combination of salinity and temperature, diatoms with an initial number of cells of 10^5^ cells/mL were exposed to each of the 6 concentrations: 0.5, 1, 3, 5, 10 or 50 mg/L of ZnO, ZnO-NPs or ZnSO_4_ in triplicate along with the control (no test chemicals). The test glass vials (10 mL in volume), each containing 6 mL of test solution, were placed in an environmental chamber maintained at the target temperature under a 14:10 h light: dark photoperiod for 96 h with a mean light intensity of 1160 lux (±80 lux; ±95% confidence interval; *n* = 20; LT Lutron LM-8000A, Taiwan). Containers were shaken regularly. After 96-h of the exposure, 500 µL of algal culture was sampled from each vial; the cell density was measured in triplicate using a cell counter (Multisizer II, Coulter, Fullerton, CA). Growth rate was calculated as *µ* = [ln(*N*′) − ln(*N*)]/*t*, where *N*′ is final cell count; *N* is initial cell count; and *t* is test period in day; it was used to determine growth inhibition compared to the control diatoms. Toxicity endpoints were the median inhibition concentrations when compared against the growth performance of the control diatoms (i.e., IC50). The IC50 values of ZnO, ZnO-NPs and ZnSO_4_ to *T*. *pseudonana* were calculated based on the particle concentrations to determine the toxic potencies of the test chemicals. The IC50 values were also calculated based on the total Zn content of test chemicals to compare toxic potencies of Zn.

### Gene expression quantification

*T*. *pseudonana* was exposed, in triplicate, to ZnO, ZnO-NPs or ZnSO_4_ by use of a factorial experimental design with 3 temperatures (10, 25 or 30 °C) × 2 salinities (12 or 32 PSU) × 2 time points (24 or 96 h). The 3 selected temperatures represented the low, optimal and high temperatures, whereas the two selected salinities represented the lesser and greater ionic strength scenarios for the algal growth. Exposure concentrations were set at the IC20 and IC50 based on results of inhibition of growth by Zn after 96 h conducted at 25 °C and 32 PSU (Table S2, SI). Tests of toxicities to diatoms were conducted in parallel with the control (without test chemicals). Test solutions (500 mL) in glass conical flasks with initial cell density of 10^5^ cells/mL were randomly placed and regularly shaken in an environmental chamber maintained at the target temperature. Cells (~10^6^–10^7^ cells/mL) were harvested by filtration (0.8-µm filter membrane; Millipore, Ireland) after 24 or 96 h of incubation. At 24 h, diatoms showed early responses to the chemical exposure (Fig. S4, SI), while duration of exposure of the growth inhibition test was 96 h.

Samples of algae were re-suspended in a 50-mL falcon tube with 15 mL filtered artificial seawater at the corresponding treatment salinity, and then centrifuged at 10,000× *g* for 3 min, collected in 1.5-mL Eppendorf tubes. Samples were briefly rinsed with autoclaved Milli-Q water and incubated in RNA*later* ^TM^ (Qiagen, Germany) at 4 °C overnight, and then stored at −80 °C until RNA extraction. The protocol of RNA extraction and gene expression analysis followed Yi *et al*.^47^. Primer sequences of selected genes are listed in Table S3, SI.

### Statistical analyses

The primary particle sizes of ZnO and ZnO-NPs dry powder were compared using a Student’s *t*-test. Four-way analysis of variance (ANOVA), followed by post-hoc Student-Newman-Keuls (SNK) multiple comparisons test (SPSS version 19; SPSS Inc., Chicago) was used to test the significance of the four fixed factors, namely water temperature, salinity, chemical and exposure concentration, and their interacting influences on aggregation, ion dissolution and zeta potential of ZnO and ZnO-NPs, respectively. Normal probability distribution of the datasets were checked by Kolmogorov-Smirnov test. Homogeneity of variance was tested with Levene’s test. When the datasets did not follow normal distribution or exhibit homogeneity of variance, they were log-transformed.

Statistical evaluations of data on inhibition of growth were done by use of GraphPad Prism 5 (GraphPad software, Inc., San Diego). Toxicity endpoints, IC50 values, of each test chemical and each temperature and salinity regime were determined by use of a sigmoidal log(agonist)-response regression model. A two-way ANOVA, followed by SNK post-hoc test (SPSS version 19), was used to compare the effects water temperature and salinity on the IC50 values calculated based on particle concentration and total Zn concentration.

Differences in patterns of expression of genes in *T*. *pseudonana* among different treatment groups were revealed using multivariate statistical analyses (PRIMER 6; Primer-E Ltd, Plymouth). All variables were normalized using Euclidean distance. Permutational multivariate analysis of variance (PERMANOVA) and analysis of similarity (ANOSIM) were conducted to infer if there were significant differences between treatments of the five different factors (i.e., temperature, salinity, chemical, exposure concentration and time point). Data were considered to be statistically different when *p* < 0.05. Heatmaps of gene expression profiles were generated by Genesis software (Graz University of Technology, Austria).

## Electronic supplementary material

Supplementary Information
