# Supplementary material for: Influences of temperature and salinity on physicochemical properties and toxicity of zinc oxide nanoparticles to the marine diatom Thalassiosira pseudonana
Source: Sci Rep. 2017 Jun 16;7:3662. doi: 10.1038/s41598-017-03889-1 (PMC5473898; doi:10.1038/s41598-017-03889-1)
Supplement: Supplementary file 1 — Supplementary Information [file 41598_2017_3889_MOESM1_ESM.pdf]

Supplementary Information

Influences of temperature and salinity on physicochemical properties and toxicity of zinc oxide nanoparticles to the marine diatom *Thalassiosira pseudonana*

Mana M. N. Yung[a], Kevin W. H. Kwok[b], Aleksandra B. Djurišić[c], John P. Giesy[a,d,e,f,g] and Kenneth M. Y. Leung[a,h]*

Number of pages: 16

Morphology and particle sizes of ZnO and ZnO-NPs

Transmission electron microscope (TEM; Tecnai G2 20S-TWIN at 200 kV, Philips, The Netherlands) was used to determine the morphology and size of ZnO and ZnO-NPs particles. Powders of ZnO and ZnO-NPs were dispersed in pure ethanol, one drop (0.7 μL) of the solution was then placed onto an ultrathin carbon-coated copper grid. All specimens were dried at room temperature before analysis. TEM images were taken by viewing five randomly selected fields. The mean size of ZnO and ZnO-NPs were calculated by measuring 100 randomly selected particles using Image J software (version 1.47, National Institute of Health, USA).

The TEM images showed particles of ZnO resembled elongated cubes whereas particles of ZnO-NPs were mainly ellipsoidal (Figure S1). Particle size of ZnO (135 ± 9 nm; mean ± 95% confidence interval) was significantly larger than ZnO-NPs (27 ± 1 nm) ($t_{0.05\,(2),\,104} = 23.28$, $p < 0.001$).

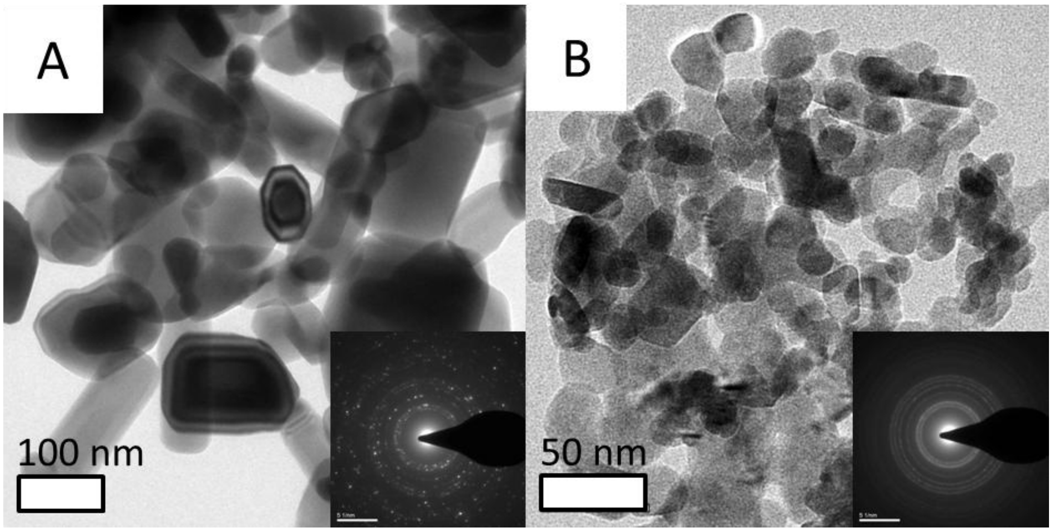

Figure S1. Morphology of: (A) ZnO and (B) ZnO-NPs dry powder. Inset: electron diffraction pattern of the particles.

Preparation of ZnO and ZnO-NPs suspensions

Autoclaved, filtered artificial seawater at five test salinities (12, 17, 22, 27 or 32 PSU; pH 8.2 ± 0.1) was prepared by dissolving appropriate amount of artificial sea salt (Instant Ocean, USA) in Milli Q water (18.2 MΩcm) followed by filtration (0.45-μm membrane filter, Millipore, Ireland).

ZnO and ZnO-NPs stock suspensions of 100 mg/L were prepared, respectively, in triplicate at 25 different combinations of the five test salinities (12, 17, 22, 27 or 32 ± 0.5 PSU) and five temperatures (10, 15, 20, 25 or 30 ± 1℃) with continuous stirring inside an orbital shaker chamber (~200 rpm; set at 10, 15, 20, 25 or 30℃ ; model 3528-1, Lab-line Instruments Inc., Melrose Park, USA) for eight days. Six test concentrations of 0.5, 1, 3, 5, 10 and 50 mg/L, which were applied as the exposure concentrations for the diatom toxicity test, were then prepared from the stock suspensions through serial dilution with filtered artificial seawater at test salinities and temperatures. A full factorial experiment design of 5 temperatures × 5 salinities × 6 concentrations was applied for physicochemical analyses. Salinity in each test suspension was checked daily using a refractometer (S/Mille, Atago, Japan), while both pH and temperature were checked daily using a pH meter equipped with a digital thermometer (Mettler-Toledo AG, Switzerland). Salinity and temperature in each treatment remained constant throughout the experiment (within ± 0.5 PSU and ± 1℃).

Zeta potential of ZnO and ZnO-NPs

Zeta potential of both ZnO and ZnO-NPs were less negative at higher temperature, showing that a rise in temperature could enhance aggregation of particle (Figure S2). Zeta potential was generally more negative when concentration increased. No obvious pattern was observed for the effect of salinity on zeta potential. Nonetheless, interactions between temperature and exposure concentration on zeta potential were significant for ZnO and ZnO-NPs (ZnO: $F_{12, 200} = 6.54$, $p < 0.001$; ZnO-NPs: $F_{12, 200} = 8.97$, $p < 0.001$).

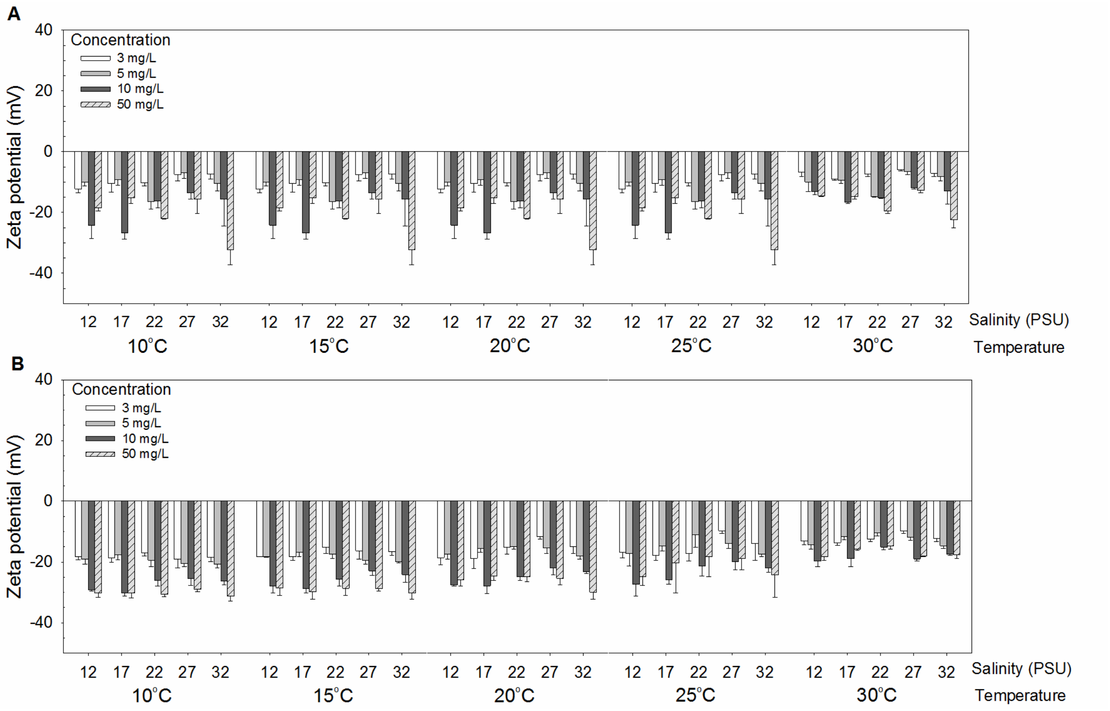

Figure S2. Mean zeta potential of: (A) ZnO and (B) ZnO-NPs at different combinations of temperature, salinity and exposure concentration after eight days of exposure (mean and 95% confidence interval, $n = 3$). Zeta potential of both particles at 0.5 and 1 mg/L were less than detection limit and such data were not shown.

Temperature and salinity effects on growth rate of *T. pseudonana*

Initial cell concentration of *T. pseudonana* culture at exponential growth phase (96 h old) was determined using hemocytometer (Neubauer Improved, Precicolor HGB, Germany) and a compound microscope (Olympus BX50, Japan) at 200 × magnification. An appropriate amount of diatom culture was added to each of the 10-mL autoclaved test glass vial (with autoclaved lids) with 8 mL autoclaved f/2 + Si medium at a combination of five temperatures and five salinities to obtain an intial algal centration of 10^5 cells/mL. Each treatment group contained three replicates. The test glass vials were placed randomly in the environmental chamber, and shaken regularly with a 14:10 h light: dark photoperiod. Algal culture (500 μL) was sampled from each vial for cell count measurements in triplicate using a cell counter (Multisizer II, Coulter, Fullerton) over the 7-day test period with daily temperature and salinity check. Growth rate was calculated as: $\mu = [\ln(N') - \ln(N)]/t$, where N' is final cell count; N is initial cell count; and t is test period in day. The growth rates of *T. pseudonana* at Day 4, where maximum growth rates were found, were reported for each treatment group (i.e., from 25 different temperature and salinity conditions).

Temperature and salinity had an interacting effect on the growth rate of *T. pseudonana* (Two-way ANOVA: $F_{16, 50} = 22.68$, $p < 0.001$). The algal growth rate at 20℃ was the fastest, followed by 25℃; diatom at 10°C and 30°C grew at a slower rate (SNK post hoc test, $p < 0.05$). Effect of salinity on the algal growth rate varied among different temperatures; at lower temperature (10°C and 15°C) the diatom grew faster at lower salinity (12 and 17 PSU) but slower at higher salinity (27 and 32 PSU); at optimum growth temperature (20°C and 25°C) the diatom grew faster at median salinity (22 PSU); at high temperature (30°C) the diatom growth rate was not significantly affected by salinity.

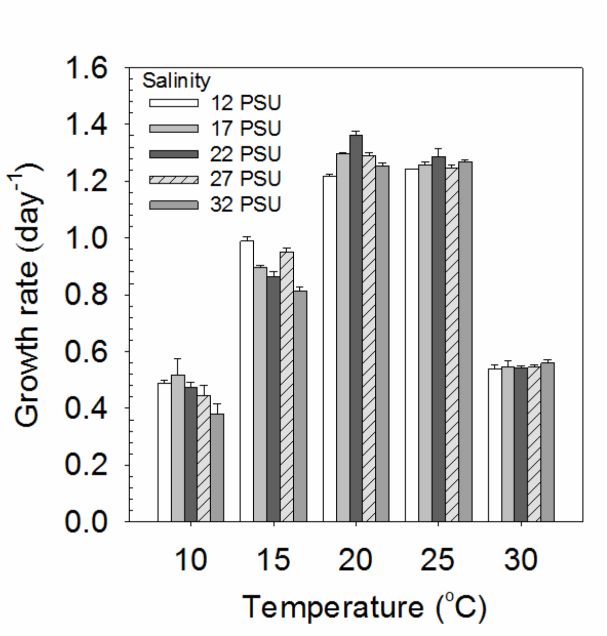

Figure S3. Growth rate of the control *T. pseudonana* (without addition of any test chemicals) at different combinations of temperature (10, 15, 20, 25 and 30℃) and salinity (12, 17, 22, 27 and 32 PSU; inserted legend) at day 4 (mean and 95% confidence interval, n = 3).

Expression of gene in *T. pseudonana* over 96 h

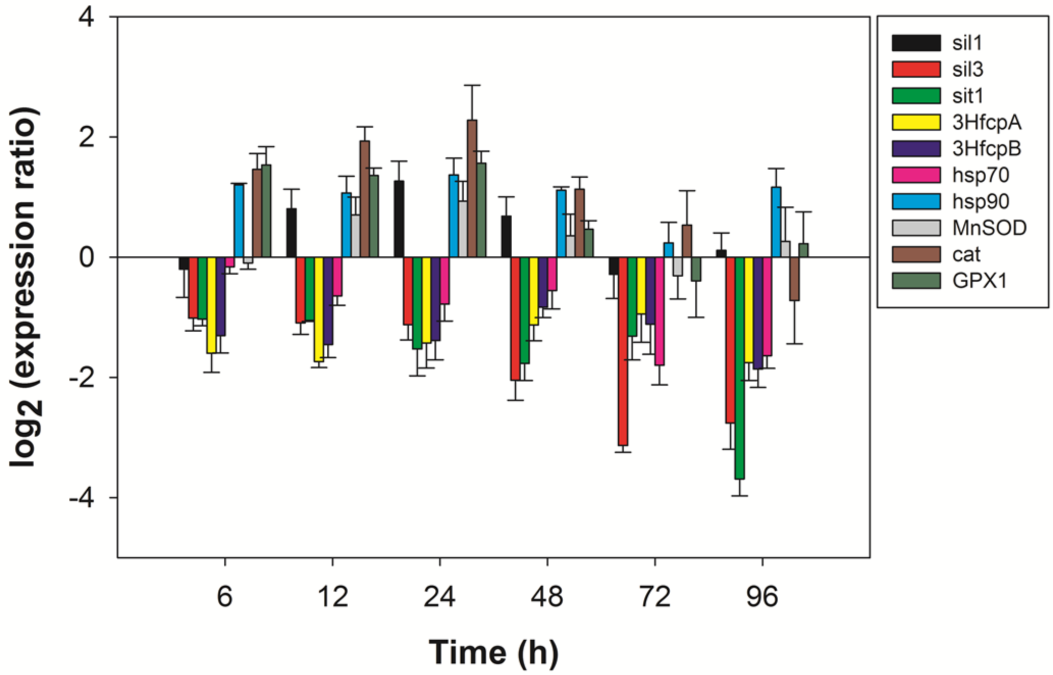

Figure S4. Expression of genes in *T. pseudonana* after exposure to ZnO-NPs (1.5 mg/L) at 25℃ and 32 PSU for different exposure duration (6, 12, 24, 48, 72 or 96 h); mean and SD (n = 3).

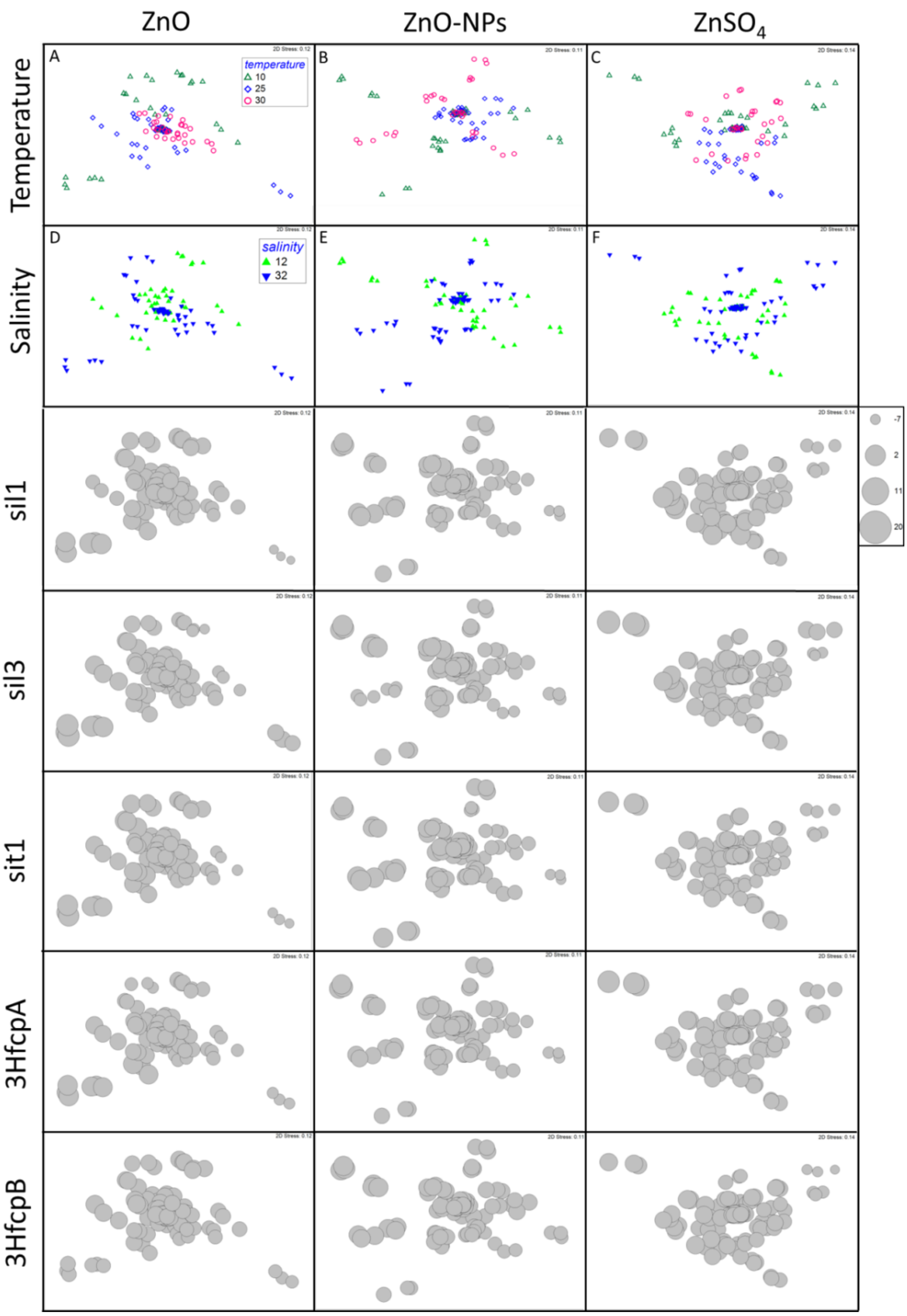

Figure S5. nMDS ordination plots of differentially expressed genes in the marine diatom *T. pseudonana* exposed to ZnO (left panels), ZnO-NPs (middle panels) and $ZnSO_4$ (right panels) at different temperatures (A-C) and salinities (D-F). The same nMDS are superimposed with bubbles showing the gene expression patterns of *T. pseudonana*. Large circles represent up-regulation of genes; small circles represent down-regulation of genes.

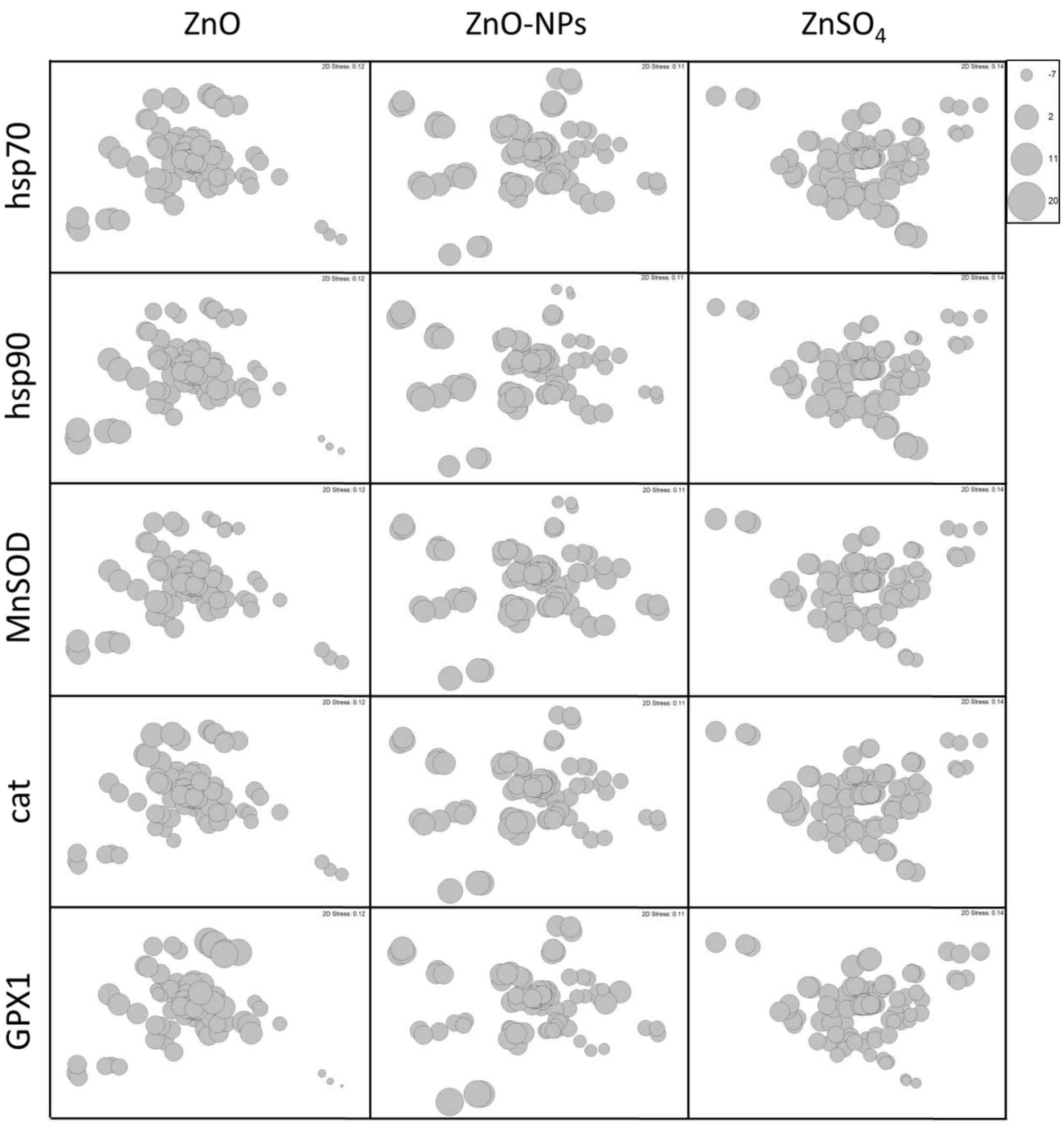

Figure S5. Continued.

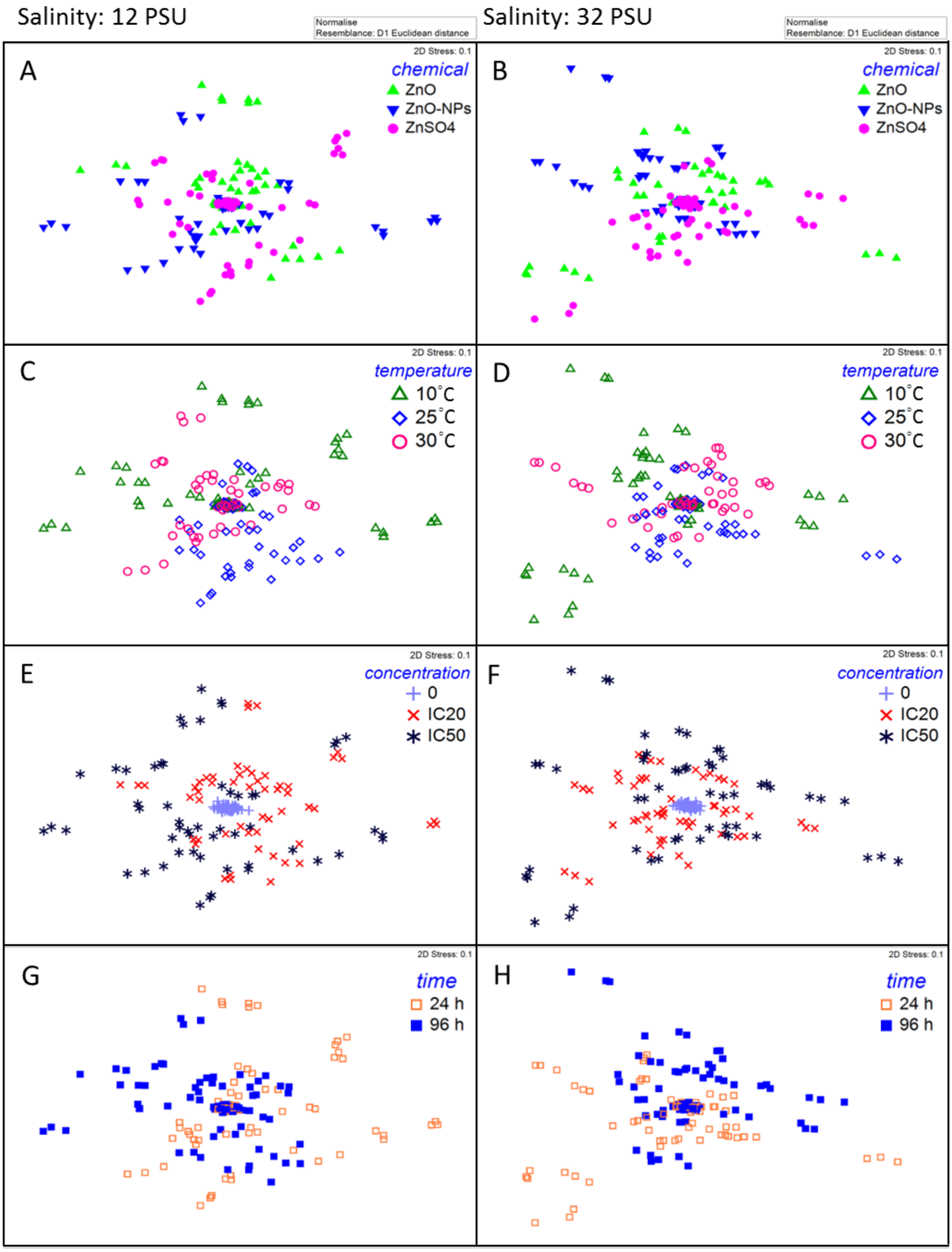

Figure S6. nMDS ordination plots of differentially expressed genes in the marine diatom *T. pseudonana* exposed to control, or various (A-B) chemicals; (C-D) temperatures; (E-F) exposure concentrations; and (G-H) time points under lesser (12 PSU; left panels) and greater salinity (32 PSU; right panels).

Table S1A. 96-h IC50 values based on concentrations of particles (95% confidence interval) of ZnO to *T. pseudonana* at different combinations of temperature and salinity based on growth inhibition relative to the controls. Values with the same letter denoted overlapping of 95% confidence interval.

96-h IC50 (mg/L of ZnO)	12 PSU	17 PSU	22 PSU	27 PSU	32 PSU
10℃	1.60 (0.20)^{ab}	1.70 (0.06)^{a}	1.80 (0.20)^{ac}	1.91 (0.02)^{b}	2.20 (0.10)^{de}
15℃	1.94 (0.05)^{bc}	1.93 (0.08)^{bc}	1.88 (0.03)^{b}	2.09 (0.02)^{d}	2.27 (0.01)^{e}
20℃	2.60 (0.10)^{f}	2.86 (0.01)^{g}	2.80 (0.10)^{fg}	2.90 (0.10)^{fg}	3.03 (0.02)^{h}
25℃	2.70 (0.20)^{fg}	3.17 (0.01)^{i}	3.22 (0.07)^{ij}	3.26 (0.07)^{j}	3.32 (0.04)^{j}
30℃	1.75 (0.04)^{a}	1.85 (0.05)^{b}	1.90 (0.10)^{bc}	1.99 (0.05)^{c}	1.98 (0.04)^{c}

Table S1B. 96-h IC50 values based on concentrations of particles (95% confidence interval) of ZnO-NPs to *T. pseudonana* at different combinations of temperature and salinity based on growth inhibition relative to the controls. Values with the same letter denoted overlapping of 95% confidence interval.

96-h IC50 (mg/L of ZnO-NPs)	12 PSU	17 PSU	22 PSU	27 PSU	32 PSU
10℃	1.30 (0.10)^{a}	1.70 (0.20)^{be}	1.70 (0.10)^{cd}	1.70 (0.20)^{be}	1.90 (0.20)^{ce}
15℃	1.78 (0.01)^{d}	1.84 (0.02)^{e}	1.89 (0.03)^{e}	1.82 (0.09)^{ce}	1.88 (0.07)^{e}
20℃	2.40 (0.20)^{fg}	2.59 (0.03)^{g}	2.67 (0.06)^{gh}	2.70 (0.10)^{gh}	2.84 (0.02)^{h}
25℃	2.38 (0.03)^{f}	2.80 (0.10)^{h}	3.00 (0.20)^{hi}	3.15 (0.01)^{i}	3.24 (0.09)^{i}
30℃	1.51 (0.03)^{b}	1.66 (0.05)^{c}	1.70 (0.10)^{cd}	1.66 (0.03)^{c}	1.69 (0.06)^{c}

Table S1C. 96-h IC50 values based on concentrations of particles (95% confidence interval) of $ZnSO_4$ to *T. pseudonana* at different combinations of temperature and salinity based on growth inhibition relative to the controls. Values with the same letter denoted overlapping of 95% confidence interval.

96-h IC50 (mg/L of $ZnSO_4$)	12 PSU	17 PSU	22 PSU	27 PSU	32 PSU
10℃	1.69 (0.04)[ab]	1.80 (0.09)[bc]	1.88 (0.02)[c]	1.90 (0.30)[ad]	1.96 (0.09)[cd]
15℃	2.05 (0.05)[d]	2.00 (0.02)[d]	2.01 (0.04)[cd]	2.32 (0.09)[e]	2.30 (0.10)[e]
20℃	2.64 (0.02)[f]	2.61 (0.06)[f]	2.79 (0.07)[g]	2.95 (0.07)[h]	3.15 (0.01)[i]
25℃	2.59 (0.07)[f]	2.87 (0.08)[gh]	3.12 (0.04)[i]	3.41 (0.08)[j]	3.49 (0.03)[j]
30℃	1.65 (0.01)[a]	1.73 (0.06)[b]	1.88 (0.04)[c]	1.91 (0.06)[c]	1.89 (0.09)[c]

Table S1D. 96-h IC50 values on the basis of total zinc concentration (95% confidence interval) of ZnO to *T. pseudonana* at different combinations of temperature and salinity based on growth inhibition relative to the controls. Values with the same letter denoted overlapping of 95% confidence interval.

96-h IC50 (mg/L of Zn)	12 PSU	17 PSU	22 PSU	27 PSU	32 PSU
10℃	1.29 (0.20)[ab]	1.36 (0.05)[a]	1.42 (0.20)[ac]	1.53 (0.02)[b]	1.73 (0.10)[ce]
15℃	1.56 (0.04)[bc]	1.55 (0.06)[bc]	1.51 (0.02)[b]	1.69 (0.02)[d]	1.83 (0.01)[e]
20℃	2.07 (0.09)[f]	2.30 (0.01)[g]	2.27 (0.10)[fg]	2.30 (0.10)[g]	2.44 (0.01)[h]
25℃	2.18 (0.10)[fg]	2.55 (0.01)[i]	2.59 (0.06)[ij]	2.62 (0.06)[j]	2.67 (0.03)[j]
30℃	1.40 (0.04)[a]	1.49 (0.04)[b]	1.50 (0.08)[bc]	1.60 (0.04)[c]	1.59 (0.03)[c]

Table S1E. 96-h IC50 values on the basis of total zinc concentration (95% confidence interval) of ZnO-NPs to *T. pseudonana* at different combinations of temperature and salinity based on growth inhibition relative to the controls. Values with the same letter denoted overlapping of 95% confidence interval.

96-h IC50 (mg/L of Zn)	12 PSU	17 PSU	22 PSU	27 PSU	32 PSU
10℃	1.07 (0.10)^{a}	1.37 (0.10)^{be}	1.40 (0.08)^{cd}	1.36 (0.20)^{be}	1.50 (0.20)^{ce}
15℃	1.43 (0.01)^{d}	1.48 (0.01)^{e}	1.52 (0.03)^{e}	1.46 (0.07)^{ce}	1.51 (0.06)^{e}
20℃	1.91 (0.20)^{fg}	2.08 (0.02)^{g}	2.15 (0.04)^{gh}	2.19 (0.10)^{gh}	2.28 (0.02)^{h}
25℃	1.91 (0.02)^{f}	2.26 (0.09)^{h}	2.44 (0.10)^{hi}	2.53 (0.01)^{i}	2.60 (0.07)^{i}
30℃	1.21 (0.02)^{b}	1.34 (0.04)^{c}	1.38 (0.08)^{cd}	1.34 (0.03)^{c}	1.36 (0.05)^{c}

Table S1F. 96-h IC50 values on the basis of total zinc concentration (95% confidence interval) of $ZnSO_4$ to *T. pseudonana* at different combinations of temperature and salinity based on growth inhibition relative to the controls. Values with the same letter denoted overlapping of 95% confidence interval.

96-h IC50 (mg/L of Zn)	12 PSU	17 PSU	22 PSU	27 PSU	32 PSU
10℃	0.68 (0.02)^{ab}	0.73 (0.04)^{bc}	0.76 (0.01)^{c}	0.78 (0.10)^{ad}	0.79 (0.04)^{cd}
15℃	0.83 (0.02)^{d}	0.81 (0.01)^{cd}	0.81 (0.02)^{cd}	0.94 (0.03)^{e}	0.94 (0.04)^{e}
20℃	1.07 (0.01)^{f}	1.06 (0.02)^{f}	1.13 (0.03)^{g}	1.20 (0.03)^{h}	1.28 (0.01)^{i}
25℃	1.05 (0.03)^{f}	1.16 (0.03)^{gh}	1.27 (0.02)^{i}	1.38 (0.03)^{j}	1.41 (0.01)^{j}
30℃	0.67 (0.01)^{a}	0.70 (0.02)^{b}	0.76 (0.01)^{c}	0.77 (0.03)^{c}	0.76 (0.04)^{c}

Table S2. Exposure concentrations of ZnO, ZnO-NPs and $ZnSO_4$ to *T. pseudonana* for gene expression study.

	Low concentration		High concentration	
	96-h IC20 (mg/L)	96-h IC20 (mg/L of Zn)	96-h IC50 (mg/L)	96-h IC50 (mg/L of Zn)
ZnO	1.8	1.4	3.3	2.7
ZnO-NPs	1.6	1.3	3.2	2.6
$ZnSO_4$	1.8	0.7	3.5	1.4

Table S3. Primer sequences used to monitor gene expression in *T. pseudonana*.

Name of the gene	Abbrevia-tion	Primer sequences (5'→3')	References/ Gene ID
Glyceraldehyde-3-phosphate dehydrogenase	*gapdh*	F: GGAGAAGGCCTCCATGCAT<br>R: TGGAGCCGAGATGACAACCT	Bopp and Lettieri (2007)
Silaffin precursor 1	*sil1*	F: CCGTCACCCTCTCCTGAAAC<br>R: ATGGGAGCAGCGGTAATGG	Bopp and Lettieri (2007)
Silaffin precursor 3	*sil3*	F: GGTGCAAAGAGTGCCAAGATG<br>R: GCTGCGTCCTCCGACTTTC	Bopp and Lettieri (2007)
Silicon transporter 1	*sit1*	F: TTGCCGAGGATGCCTAAACTT<br>R: TGACGAGCTACTGCAGGTTCA	Bopp and Lettieri (2007)
Fucoxanthin-chlorophyll a/c light harvesting protein	*3HfcpA*	F: CTCCCTCCAGGTTCCTGTTG<br>R: AGCGAGCTCAAGGAATCCAA	Bopp and Lettieri (2007)
Fucoxanthin-chlorophyll a/c light harvesting protein	*3HfcpB*	F:AGTTCGATGAGGAGACCAAGCT<br>R: GGCACGTCCGTTGTTCAAC	Bopp and Lettieri (2007)
Heat shock protein	*hsp70*	F: GGATGTGACTCCCTTGAGTTT<br>R: GGGATAGCAGTGTTCCTCTTAAT	GI:224001715
Heat shock protein	*hsp90*	F: AGGCTCTTACGGCCGGGGCGGA<br>R: AAGACCCGCCAGCCTCGGAAGCC	Shi et al. (2013)
Manganese superoxide dismutase	*MnSOD*	F: ATGAAAATCCATCATGATAAGCAT<br>R: TCCTCGCACGGGGACTCCTG	Wolfe-Simon et al. (2006)
catalase	*cat*	F: GTTGATGATTCGGTTGGCTTGGC<br>R: AGTTGAGAGGTGCAAGACGGATG	Davis et al. (2006)
Glutathione peroxidase	*GPX1*	F: CAAAGGCGACGTGCTATGCGTC<br>R: GGCTCCTGAGCTCCAAACTGATT	Davis et al. (2006)

References

Bopp, S. K. & Lettieri, T. Gene regulation in the marine diatom *Thalassiosira pseudonana* upon exposure to polycyclic aromatic hydrocarbons (PAHs). *Gene* **396**, 293-302 (2007).

Davis, A. K., Hildebrand, M. & Palenik, B. Gene expression induced by copper stress in the diatom *Thalassiosira pseudonana*. *Eukaryotic Cell* **5**, 1157-1168 (2006).

Shi, X., Gao, W., Chao, S. H., Zhang, W. & Meldrum, D.R. Monitoring the single-cell stress response of the diatom *Thalassiosira pseudonana* by quantitative real-time reverse transcription-PCR. *Appl. Environ. Microbiol.***79**, 1850-1858 (2013).

Wolfe-Simon, F., Starovoytov, V., Reinfelder, J. R., Schofield, O. & Falkowski, G. Localization and role of manganese superoxide dismutase in a marine diatom. *Plant Physiol.* **142**, 1701-1709 (2006).
